# New Seasonal Shift in In-Stream Diurnal Nitrate Cycles Identified by Mining High-Frequency Data

**DOI:** 10.1371/journal.pone.0153138

**Published:** 2016-04-13

**Authors:** Alice H. Aubert, Lutz Breuer

**Affiliations:** Institute for Landscape Ecology and Resources Management (ILR), Research Centre for BioSystems, Land Use and Nutrition (IFZ), Justus Liebig University Giessen, Heinrich-Buff-Ring 26, Giessen, Germany; CAS, CHINA

## Abstract

The recent development of *in-situ* monitoring devices, such as UV-spectrometers, makes the study of short-term stream chemistry variation relevant, especially the study of diurnal cycles, which are not yet fully understood. Our study is based on high-frequency data from an agricultural catchment (Studienlandschaft Schwingbachtal, Germany). We propose a novel approach, i.e. the combination of cluster analysis and Linear Discriminant Analysis, to mine from these data nitrate behavior patterns. As a result, we observe a seasonality of nitrate diurnal cycles, that differs from the most common cycle seasonality described in the literature, i.e. pre-dawn peaks in spring. Our cycles appear in summer and the maximum and minimum shift to a later time in late summer/autumn. This is observed both for water- and energy-limited years, thus potentially stressing the role of evapotranspiration. This concluding hypothesis on the role of evapotranspiration on nitrate stream concentration, which was obtained through data mining, broadens the perspective on the diurnal cycling of stream nitrate concentrations.

## Introduction

High-temporal resolution or high-frequency water chemistry time series, such as a stream’s nutrient concentration, are increasingly interesting because (i) they question current sampling strategies regarding the control of water quality [1, 2], (ii) they allow for the reduction of the uncertainty related to flux estimation [3], and (iii) they are expected to give new insights at catchment functioning [4] as well as, most importantly, river ecosystems functioning [5].

Only recently is high-frequency (i.e. hourly or even higher) data acquisition of stream solutes possible at relatively reduced effort and costs in terms of working force and time, over extended periods (up to years) and at low solute concentrations [6, 7]. Hyperspectral UV-spectrometers allow data to be collected at sub-hourly resolution, thereby providing a time series of hydrochemistry with the same resolution as high-frequency hydrological or meteorological observations [8, 9].

Specifically, high-frequency water quality data are useful to address the unresolved issue of the diurnal cycles of stream nitrate concentration [10–17]. Most studies have used data spanning repeated short periods, with many variables that also focus on stream diurnal nitrate variations. Among these studies, those focusing on headwater catchments, which are mostly forested, have reported a “pre-dawn peak” in nitrate concentration, i.e., a maximum concentration at dawn when the water experiences darkness for the longest time and no photo-mediated processes occur [14, 15, 18]. The drivers of diurnal cycles are stream biological activity, such as assimilation by primary producers [19], which is often controlled or limited by other drivers such as light, temperature, riverine oxygen dynamics or pH [6, 10, 17]; and/or time-varying physical transport [13], e.g., through the stream bed sediment, or the hyporheic and riparian zones.

However, the same studies have reported inconsistent diurnal cycles throughout the year in forested headwater catchments. Cycles are mostly observable under hydrologically stable conditions and during biologically active periods, i.e., at low flow in early spring before leaf growth when solar radiation reaches the streams. Other explanations for the seasonal change in the diurnal cycles are seasonal changes in in-stream residence times or varying day lengths [20].

The present paper analyzes a large high-frequency dataset with data mining approach, i.e. with data analysis aiming to extract patterns from our large dataset, to identify nitrate diurnal cycles and associated potential drivers in the hydrology, soil and atmosphere. It contributes to the few studies on diurnal cycles conducted in human-impacted catchments [13, 21, 22]. We address the following questions: Can we find diurnal cycles in our high-frequency nitrate time series, and are they stable over time? What do these cycles look like? What are their primary drivers?

## Materials and Methods

### Schwingbachtal study site

The Vollnkirchener Bach catchment is part of the Schwingbachtal landscape observatory (references and data are available at http://fb09-pasig.umwelt.uni-giessen.de:8081/), located in central Germany, in the federal state of Hessen [23, 24]. Monitoring of hydrology, meteorology and stream chemistry started in 2008. The climate is temperate. The specific discharge for the hydrological year 2012–2013 was 104.5 mm y^-1^, and for 2013–2014, it was 64.5 mm y^-1^.

The 3.7 km^2^ catchment is drained by a 4.7-km-long 2^nd^ Strahler order stream. Forested land covers 48% of the catchment in the east and south, arable land covers 35% in the west and grassland covers 9%, primarily along the stream. The remaining 8% includes infrastructure from settlements. Land use follows the soil distribution: forested land is primarily situated on Cambisols, agriculture is on Stagnosols with thick Loess layers (Stagnic Luvisols), and grasslands are found on Gleysols.

The catchment topography is gentle. The elevation ranges from 235 m above sea level at the outlet in the north to 351 m a s l in the south. The geology of the southern part (the headwater) consists of limestone, sandstone and quartzite and provides large groundwater storage capacity [23]. The northern part of the catchment lies on clay shale.

### A rich data set

The analyses are based on hydrological, meteorological and high-frequency stream nitrate concentration data for the hydrological years 2012–2013 and 2013–2014. The nitrate concentrations were measured using a hyperspectral UV-spectrometer (ProPS, Trios, Rastede, Germany, wavelength range 200–360 nm, pathlength 5 mm, solar power supplied) beginning in March 2012. The ProPS was coupled with an airblast system, which was turned on for 5 seconds before measurement to clean the lens. The lens was also cleaned weekly with acetone using a specific lens handkerchief because the airblast was not sufficient to limit biofouling, as we had observed in the test period of the sensor typical drift of ProPS values: continuous increase over several weeks, not reaction to storm event, and unmatching manual samples analyzed in the lab. Absorption spectra were measured every 15 min. The global pre-calibration by the manufacturer was modified to fit the local water matrix, as described by the manufacturer [25], and in other work [26], using a linear function and data collected during a test period of one month. The wavelength area of 200–220 nm was then used to calculate the nitrate concentration. Both the spectra and concentration were considered for data verification. Weekly manual samples analyzed in the lab by ion chromatography (DX-120, Dionex Corporation, CA, USA) were used to check the data (detection limit: 0.1 mg l^-1^ for NO_3_^-^). All data points with a critical spectrum (spectral error (metadata for each measure) above the 0.05 Absorbance Unit threshold recommended by the manufacturer, absorbance spectrum presenting abnormal shape (visual check, controlled by recalculation of the nitrate concentration)) or concentration (random outliers, measure out of the detection range) were discarded from the present analyses. Weekly manual sampling helped identifying drift periods (due to biofouling for instance). Device malfunction and data verification reduced the time span of our data to two periods– 05 March 2013 12:45 to 24 September 2013 12:30 (10,569 data points) and 27 April 2014 00:00 to 23 October 2014 13:15 (16,566 data points)–which are, respectively, referred to as “2013” and “2014”.

The time series for the other variables, though they are longer, were reduced to the periods when nitrate concentrations were available. Discharge at the gauging station was obtained through 5-min measurements of the stream’s headwaters by pressure transducers (Diver DCX, Schlumberger Water Services, ON, Canada) installed in a flume (RBC-flumes, Eikelkamp Agrisearch Equipment, Netherlands). Weekly scale readings of the flume were used to control the Diver data. The Diver also measured water temperature. A climate station (Campbell Scientific Inc., CR1000 data logger, UK) 4 km northeast from the outlet recorded air temperature, wind speed and direction, relative humidity, solar radiation every 5 min and rainfall intensity. The sensor measuring solar radiation was defective for one week in 2013. Groundwater depth data of one piezometer were considered and measured by a Diver every 10 min. Soil moisture and temperature at 0.1 m depth were measured hourly by electromagnetic induction (5TE sensors, EM50 data logger, Decagon, Labcell LTD) beginning 14 June 2013.

### Data analysis

Because 2013 and 2014 differed greatly with regard to meteorological boundary conditions, the analyses were conducted year by year. To remove the long-term variation (seasonal trend) that might alter the results in the short term, we subtracted a smoothed time series from the raw nitrate time series. The smoothed time series was obtained by calculating a centered 1-day moving median (“rollapply” function, zoo package, R). The residual time series was then divided into days (from 00:00 to 23:45), because we focus on mining diurnal patterns, and smoothed by the centered rolling median across five data points to minimize the effect of outliers. Punctual missing data were populated with the “last observation carried forward” procedure. Days with gaps longer than 2 hours 30 minutes (10 consecutive missing data points) were discarded. We applied k-means clustering on the remaining days to identify common patterns in the diurnal variation of the stream nitrate concentration (“cascadeKM” function, Vegan package, R, using Calinski criterion). We tested clustering for k (number of clusters) ranging from 3 to 20. We visually verified the best partition; for both years three clusters contained many individuals/days and were kept for further analyses. The other clusters contained only a few days that represented unusual variations, which were not within the scope of the present study. The days belonging to the main clusters were characterized according to 12 variables or potential drivers (Table 1). Two days in 2013 and four days in 2014 were further discarded because they represented outliers for one of these 12 drivers. Each cluster, which was obtained solely using the nitrate concentration time series, was characterized by the distribution (boxplots) of the values for these 12 variables. Overall, we analyzed 134 days in 2013 and 157 days in 2014.

**Table 1:** Variables potentially driving the in-stream nitrate cycle, or potential drivers.

Variable	Unit	Driver of the nitrogen cycle
Daily amplitude of air temperature	°C	Plant growth rate; evapotranspiration
Daily amplitude of soil temperature	°C	Soil microbial activity and nutrient turnover
Daily amplitude of stream temperature	°C	Growth rates of algae and other aquatic living organisms; nitrification rate
Daily mean of solar radiation	W m^-2^	Evapotranspiration; photosynthesis
Daily maximum of solar radiation	W m^-2^	Evapotranspiration; photosynthesis
Daily mean of soil moisture	M^3^ m^-3^	Soil microbial activity and nutrient turnover (wetting pulse following dry phase[27])
Daily mean of groundwater depth	m	Groundwater is the primary contributor to discharge in Vollnkircherbach[24]
Daily standard deviation in discharge	l s^-1^	Integrative parameter reflecting overall hydrological processes
Daily maximum of rainfall intensity	mm d^-1^	Indicator for events; rainfall triggers discharge and soil moisture pulses; affects wash out of nutrients and suspended sediment concentration
Daily sum of rainfall	mm d^-1^	Indicator for long term condition; rainfall triggers discharge and soil moisture; affects plant growth
Number of previous days without rain	-	Describes droughts and potential build up of organic substrate in the catchment area
Mean daily difference between stream and air temperature	°C	Temperature difference is a proxy of potential heat exchange between air and stream

Then, we conducted a Linear Discriminant Analysis (LDA) (“lda” and “greedy.wilks” functions, MASS and klaR packages, R, respectively) to verify our descriptive understanding of the clusters. A stepwise forward variable selection using the Wilk’s Lambda criterion indicated which variables allowed for the best fitting classification of days in the clusters that were previously defined using only nitrate. Seven uncorrelated potential drivers (of 12) were used to predict the clusters based on a correlation matrix, and independence was verified (with the Variance Inflation Factor (which provides a measure of how severely the variance of an estimated regression coefficient is increased due to collinearity, if VIF is more than 5 multicollinearity is strong), fmsb package, R).

## Results

### Mining days with a diurnal cycle

**Fig 1:**
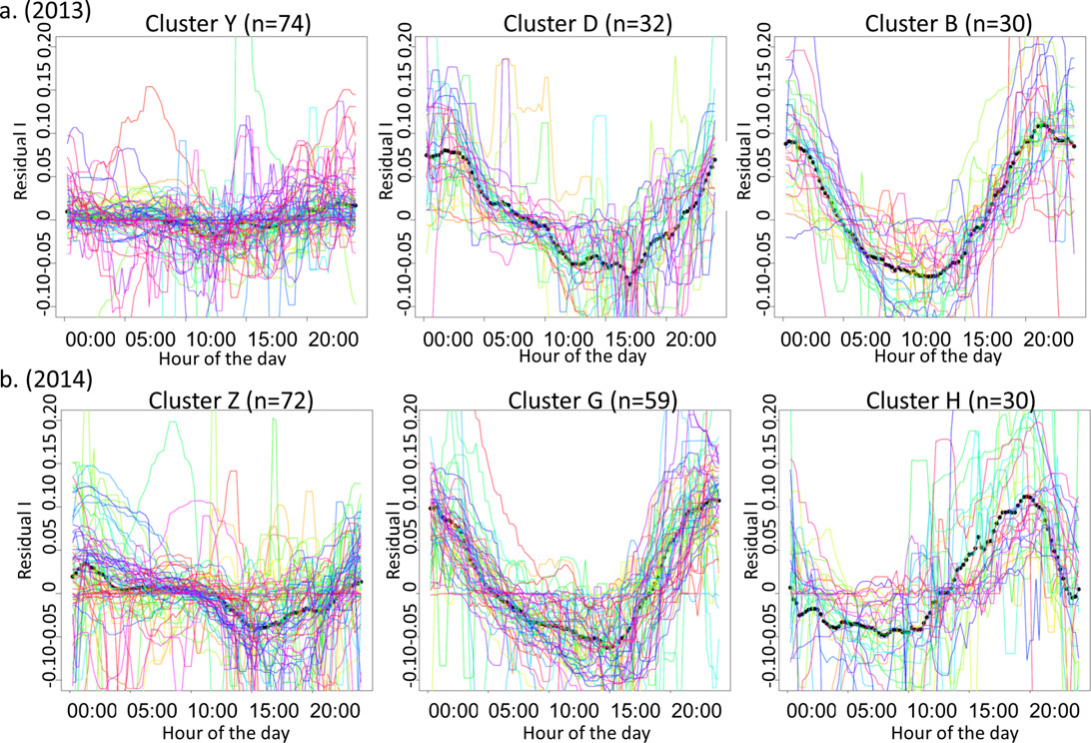
**Primary clusters for 2013 (a) and 2014 (b).** Names of the clusters are arbitrary. The black dotted line is the mean diurnal pattern. Each thin colored line represents one day. X-axis are hours (local time). Y-axis are detrended, smoothed nitrate concentrations, referred to as residual I.

The best k-means partition suggested 10 and 8 clusters for 2013 and 2014, respectively. However, only three clusters per year (Fig 1) contained a sufficient number of days to conduct further statistical analyses (numbers of days in each cluster for 2013: 74, 32, 30, 5, 5, 3, 2, 1, 1, 1; for 2014: 72, 59, 30, 3, 3, 3, 2, 1).

In 2013, among the three main clusters, clusters B and D appeared to correspond to diurnal cycles, with a 5 h lag between them. The minimum of the mean cycle was reached at 11:45 and 16:45 for clusters B and D, respectively. The maximum of the mean cycle was reached at 21:15 and 01:30 for clusters B and D, respectively. The mean pattern of cluster Y was relatively flat. It included days with no pronounced diurnal cycle.

In 2014, clusters G and H depicted diurnal cycles with amplitudes in the same range as in 2013. The mean cycle shape of cluster G was similar to that of cluster B in 2013. The minimum occurred at 14:30, and the maximum at 23:15. The shape of cluster H differed; it depicted a plateau around the minimum from 03:45 to 10:00 and a distinct maximum at 19:30. The mean pattern of cluster Z had a minimum in the afternoon at 15:00 and a maximum at night at approximately 01:00. However, considering the cloud of daily curves, it appeared that cluster Z gathered not only days with diurnal cycles of very low amplitude but also days with more erratic variations. We therefore consider cluster Z to be similar to cluster Y because they have no clear diurnal cycle.

### Background hydro-meteorological conditions of each cluster

**Fig 2:**
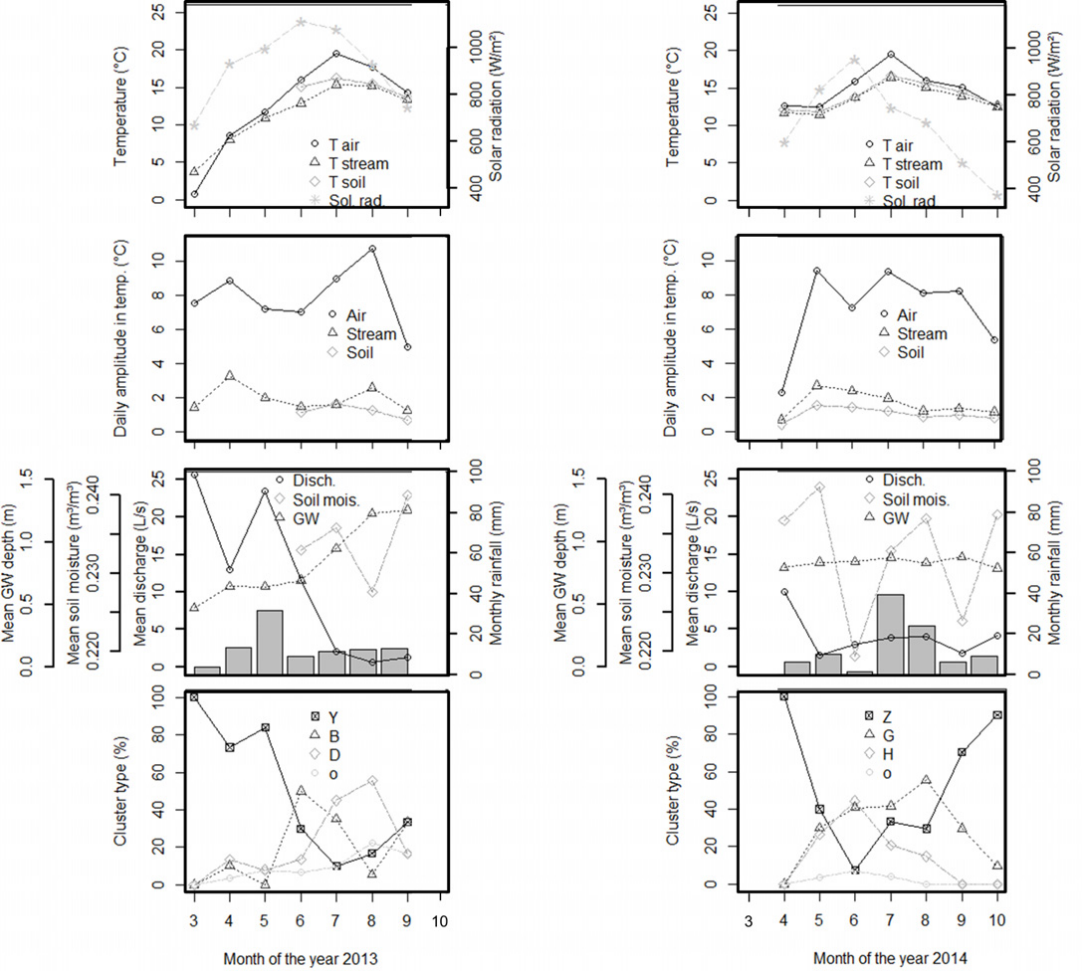
Time-series (monthly aggregation) of the hydrological and meteorological variables, showing the difference between 2013 and 2014. The last panels in each column represent the relative occurrence of each cluster (“o” being the outliers).

The years 2013 and 2014 differed in terms of the hydrology and meteorology (Fig 2). In 2014, the groundwater depth and soil moisture were approximately stable, the air temperature was above 10°C for all months, solar radiation was lower and the air temperature amplitude changed less than in 2013. In 2013, the discharge was higher and more variable throughout the year compared with 2014.

Among the 12 potential drivers (Table 1), the air, soil and stream daily amplitudes of temperature and solar radiation were correlated, as were the daily sum and maximum rainfall. Thus, we retained the following potential drivers for further analyses: amplitude of air temperature, mean soil moisture, groundwater table depth, maximum solar radiation, number of previous days without rain, maximum rainfall intensity and discharge standard deviation. The Variance Inflation Factor was less than 5 for all drivers for both years (2.01 and 2.28 for 2013 and 2014, respectively), thus ensuring independence of the drivers.

**Fig 3:**
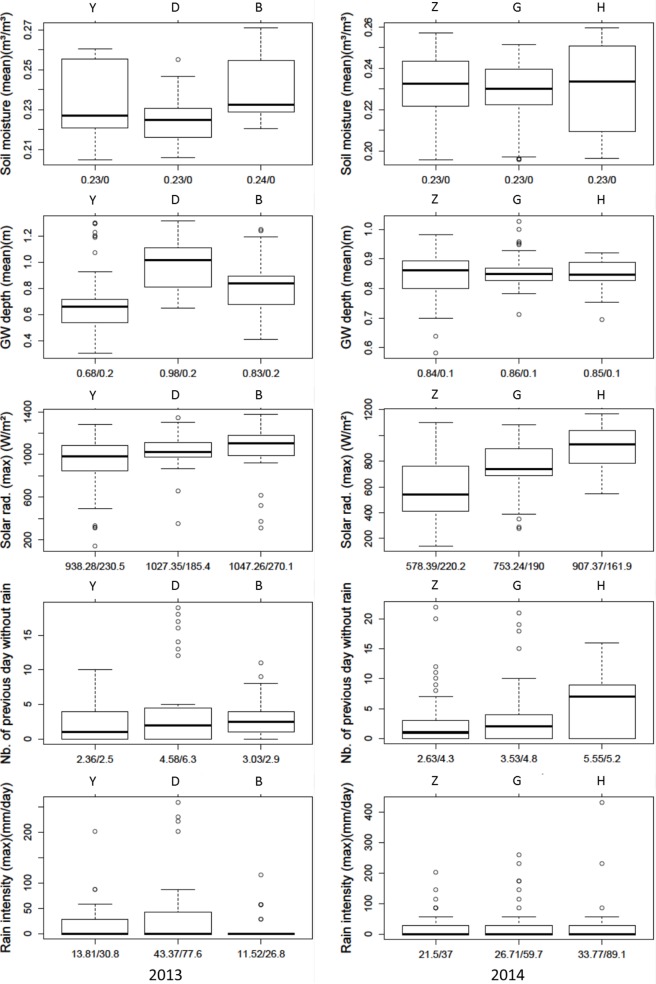
Boxplots of five potential drivers for each cluster. The left and right columns are, respectively, 2013 and 2014. Numbers below the graphs are the mean/standard deviation.

Each cluster matched specific hydrological and meteorological conditions (Fig 3). Cluster Y (“no diurnal cycle”, 2013) was characterized by a low daily air temperature of less than 4° and a shallower groundwater table. Cluster D occurred under the driest conditions of 2013, with the lowest soil moisture content and deepest groundwater table. Cluster B appeared to be intermediate.

Background hydro-meteorological conditions for 2014 differed substantially from 2013 clusters, as already mentioned with the half to double specific discharge. A lower air temperature and a lower maximum solar radiation (though highly variable) characterized cluster Z. Cluster H occurred on days with the highest air temperature and the maximum solar radiation and discharge variability. Cluster H often developed after more than 5 days without rain. Cluster G was intermediate.

We also checked the temporal relative occurrence of each cluster throughout the season (Fig 2). In 2013, cluster Y (no clear diurnal pattern) was dominant at the beginning of the season. From June onwards, clusters B and D developed with a shift in the maximum from early evening (B) towards later at night (D). In 2014, daily cycles (clusters G and H) appeared earlier in the year; in May, these clusters replaced the previously dominant cluster Z. Again, we observed a shift from an early evening maximum (H) in June to a midnight maximum (G) in August.

### The primary drivers

To check our visual descriptive understanding of the clusters and define the main drivers, we conducted a LDA (Table 2). In 2014, the maximum solar radiation alone explained the partition of the daily cycles. In 2013, the most important drivers for the daily cycles were groundwater depth and soil moisture. Because soil moisture data were not available at the start of the sampling period in 2013, we estimated an alternative model and identified the rainfall intensity and number of previous days without rain as a second and third important driver, after groundwater depth. Overall, we found 2013 to be characterized by moisture-related parameters, whereas 2014 was energy driven. The prediction error, indicating the proportion of false classification of days when using a linear combination of the explanatory variables, was improved from 49% when considering the seven variables to 45% when selecting only the significant variables for 2014. F 2013, the models using only the significant variables did not perform as well as the complete set of drivers (34% when modelling with the seven variables compared to 35% for the model of run 1 and 38% for the model of run 2).

**Table 2:** Drivers selected to explain the clusters, using Wilk’s Lambda criterion.

Variable	Wilks.lambda	F.statistics.overall	p.value.overall	F.statistics.diff	p.value.diff
**2014**					
Solar rad. (max)	0.718	29.32	1.83e-11	29.32	1.83e-11
2013, run 1 *					
GW depth (mean)	0.798	6.85	2.23e-03	6.85	2.23e-03
Soil moisture (mean)	0.622	7.10	4.22e-05	7.47	1.37e-03
2013, run 2 *					
GW depth (mean)	0.759	20.29	2.22e-08	20.29	2.22e-08
Rain intensity (max)	0.691	12.89	1.45e-09	6.27	2.52e-03
No. of previous days without rain	0.605	12.00	7.42e-12	8.98	2.25e-04

The procedure aims at explaining and predicting the cluster (class) membership of one day (individual) by stepwise forward variable selection and testing of the goodness of prediction for the overall model (F.statistics.overall: approximated F-statistic for the so far selected model and its significance test (p.value.overall)) and for each added variable in the model (F.statistics.diff: approximated F-statistic for comparing the model including the new variable with the model not including it and its significance test (p.value.diff)).

* Due to the installation of the electromagnetic induction sensor during 2013, run 1 includes soil moisture and considers data from 14 June 2013; run 2 completely covers our sampling period without considering soil moisture.

## Discussion

### Diurnal cycles differ from other headwater catchments

Unexpectedly, diurnal cycles in the Vollnkirchener Bach differed from the “pre-dawn peak” cycles described in other headwater catchments. Our maxima occurred much before dawn, between 19:30 and 01:30, which is closer to what is observed in the moorland catchment of Upper Hafren [28] and the much bigger mixed land use catchments of San Joaquin or Ichetuknee in California and Florida, respectively [10, 13]. In Sleepers Rivers, during the snow-melt season, diurnal cycles also differ from pre-dawn peaks [18], because cycles persist after snow had melted, they expect in-stream biological processes to drive the cycles. Cohen et al. [10] have observed the diurnal cycle fractionation of isotopes and have concluded that the diffusion of nitrate into the benthic sediment, the length of which depends on stream oxygen dynamics, controls the diurnal variation in denitrification. Provided that the Vollnkirchener Bach flows on loamy deposits over sand [23], this explanation is plausible in our case study. Pellerin et al. [13] have suggested that late-day minima in nitrate concentration, with timing similar to cluster D, are synchronized with peaks in chlorophyll-a and are thus algal uptake dependent. Halliday et al. [28] also strongly suggested this in-stream N uptake processes, calling for the photosynthetic assimilation by diatom biofilms and bryophytes. Observing biofouling on the ProPS device, we can affirm that as the stream gets warmer and flows more slowly, there is a bloom of in-stream vegetation. Algal uptake could therefore, explain cycles from cluster D. Our diurnal cycles clearly were asymmetric: nitrate concentrations needed more time to decrease than to increase. This supports the hypothesis of activated denitrifying biological processes starting at night, such as the process described by [29], and a relatively rapid return to baseline concentration when the processes are inactivated. But this characteristic could also simply be explained by the dispersion effect: a pure sine wave imposed on a stream will result in a skewed downstream signal as the lead part of the signal is dispersed less than the lagged part.

### Shifts in diurnal cycles throughout the season

In our catchment, the diurnal cycles were present from May/June to August, with a delayed cycle in late summer. In 2013, the diurnal cycles of cluster B primarily occurred in June and those of cluster D occurred in July and August. The diurnal cycle shifted by approximately 4 to 5 h (minimum and maximum of the cycle, respectively). In 2014, cluster Z (no clear diurnal cycle) occurred at the beginning of the sampling period, and then clusters G and H developed in May. Cluster H peaked in June, and cluster G peaked in July and August. In September and October, cluster Z took over again. The diurnal cycle shifted by at least 4 h for the minimum and maximum of the cycle. A time-shift in the diurnal cycle throughout the year was expected because (i) metabolisms are known to undergo substantial differences between spring and fall[19] (though it is expected to have a greater impact on a seasonal amplitude change of the cycle (not observed here) rather than on the timing of the cycle); (ii) the water source composition changes throughout the year[20]; and (iii) the contributions of water sources vary proportionally throughout the season [30], some sources becoming totally disconnected [31, 32]. This later point is the most plausible. However, surprisingly, our shift differed from other observations of headwater catchments. Reported diurnal cycles in forested catchments are observed in spring, when solar radiation reaches the stream before leaves grow. In spring, in the Vollnkirchener Bach, light and, therefore, photosynthetic in-stream activity did not directly induce daily cycles, particularly in 2013.

### Seasonal pattern of diurnal cycles and riparian drivers

The hydro-meteorological conditions of 2013 and 2014 differed, which resulted in contrasted boxplots and LDA variable selections. Overall, 2013 appeared to be water-limited. Model simulations highlight the dryness effect on denitrification. If less groundwater feeds the stream, for example, during the dry season, the hyporheic zone presents increased residence times and and bigger reactive fringe where denitrification can occur [33]. Others have attributed increased denitrification rates to a low flow velocity: a slow flow of water over the streambed sediment promotes deeper and longer advective paths in the sediment where denitrification occurs [34], leading to a greater portion of the total stream flow interacting with hyporheic surfaces. In other words, the dryer it is, the longer the “exposure/contact time” to denitrifying conditions [35], potentially creating diurnal cycles. However, 2014 was not water-limited and the groundwater depth was relatively constant, thus maintaining a humid vadose zone. 2014 appeared to be energy-limited (solar radiation drove the partition of clusters).

The discussion can therefore be generalized: how can similar seasonal patterns of diurnal nitrate cycles throughout the year (no cycle in spring and the appearance of a diurnal cycle in summer that shifts in time) be explained with different environmental drivers?

An answer may be that the primary drivers of 2013 and 2014 are actually proxies of a third driver, e.g., evapotranspiration (ET). 2013 was water-limited, whereas 2014 was energy-limited, but the amounts of both water and light control ET. Water stress in 2013 and energy stress in 2014 could have affected biomass production equally, leading to a comparable transpiration level. Preliminary studies of diurnal cycles in the discharge of the Vollnkirchener Bach (not presented for clarity) showed a timing typical of evapotranspiration control, as observed by others [36, 37]. Thus, we think that transpiration from the riparian zone vegetation drives the seasonal pattern of diurnal nitrate cycles throughout the year: as the summer passes, the vegetation biomass is generally more important, leading to higher ET, i.e. higher water uptake by the plants during the day, thus strengthening the importance of diurnal cycle in late summer (compare to spring). However, to confirm this hypothesis, we lacked of highly relevant time series of biological indicators. For example, dissolved oxygen, chlorophyll-a, other nitrogen forms, sap flow of riparian vegetation, or stable water isotopes would have allowed for a better assessment of the controlling biological processes.

## Conclusion

Data mining requires comprehensive data sets [38]. We used high-frequency in-stream nitrate concentrations and twelve associated environmental variables for two growing seasons in 2013 and 2014, with each season spanning more than 150 days. Such a dataset is ideal for data mining, even though some variable were strongly correlated which reduced the number of associated environmental variables to seven independent variables. We found diurnal cycles and temporal shifts throughout the season using automatic statistical procedures, which are unique to the headwater catchment and not visible in the raw time series. In our catchment, we hypothesized that ET, via riparian plant production, controls the seasonal pattern of these cycles. More specific time-series are all the more needed as any time-series are elusive, i.e. are spatially uneven convolutions of processes. Overall, we consider data mining of high-frequency stream solute concentrations to be a promising tool.

## Notes

Data following the quality check can be found at doi:10.1594/PANGAEA.859204 (https://doi.pangaea.de/10.1594/PANGAEA.859204). Raw data and complete metadata are available at http://fb09-pasig.umwelt.uni-giessen.de:8081/

## Abbreviations

ET: evapotranspiration
LDA: linear discriminant analysis
UV: ultra-violet
